# Analysis of Genetic Diversity and Population Structure of Tarim and Junggar Bactrian Camels Based on Simplified GBS Genome Sequencing

**DOI:** 10.3390/ani13142349

**Published:** 2023-07-19

**Authors:** Weikun Tao, Lazat Aniwar, Azat ZuliPicar, Hanikzi Tulafu, Rongyin Zhang, Bo Liu, Weiwei Wu, Juncheng Huang

**Affiliations:** 1 Institute of Animal Science, Xinjiang Academy of Animal Sciences, Urumqi 830000, China; taoweikun@hotmail.com (W.T.);; 2 Institute of Quality Standards, Xinjiang Academy of Animal Husbandry Sciences, Urumqi 830000, China

**Keywords:** Bactrian camel, genetic diversity, group structure, cluster analysis, functional gene selection

## Abstract

**Simple Summary:**

Camels have a high tolerance to hunger, thirst, cold, and drought, as well as the ability to travel over long distances. They are indispensable animals for agricultural production in desert areas in China. Old World camels have served humans in uses such as cross-continental caravans; transporting people and goods; connecting different cultures; providing milk, meat, and wool; and serving as draught animals. In view of the severe reduction in Bactrian camel germplasm resources, people now realize that it is particularly important to scientifically evaluate and protect the genetic integrity and diversity of native species. At the same time, there is a growing demand for intensive and sustainable meat and milk production. Using modern genomic tools for selection to improve milk and wool production is warranted. In this study, GBS technology was used to analyze the genetic diversity and genetic structure of Tarim and Junggar camels, and identify the genes important in their evolution. On this basis, the effects of resource conservation were evaluated by analyzing the differences between individuals and populations at the genome level. Screening candidate genes that may be responsible for the biological characteristics of Bactrian camels will aid research into genetic resource protection, important economic traits, and the mechanisms underlying biological characteristics, molecular breeding, and disease.

**Abstract:**

In view of the severe reduction in Bactrian camel germplasm resources, scientific evaluation, protection, and utilization is particularly important. Therefore, it is necessary to investigate the genetic diversity and genetic structure of this species, and identify the genes that have played important roles in its evolution. In this study, 21,971 SNPs were identified in 118 domestic Bactrian camels from the Tarim (*n* = 60) and Junggar (*n* = 58) populations using simplified GBS genome sequencing. The results show that Tarim and Junggar Bactrian camels have high nucleotide diversity. A phylogenetic tree constructed using structural analysis, principal component analysis (PCA), and the adjacency method (NJ) showed that Tarim and Junggar Bactrian camels were clustered together. The selection signals revealed that the Tarim and Junggar Bactrian camels shared 108 genes under positive selection, including WNT1, WNT10B, CD14, SEC61A2, DPAGT1, FOXO6, etc. These selected genes were widely involved in the immune system, embryonic development, lipid metabolism, and other processes. From a genomic analysis perspective, the genetic relationship between TLM and ZGE camels is close, with an average Fst of 0.048 and a relatively low average differentiation coefficient between the two populations. In addition, shared selected genes in the long-term depression pathway were significantly enriched in Tarim and Junggar. These findings will offer support and assistance for the exploration of genetic resource preservation, economically significant traits, and the mechanisms underlying biological characteristics, molecular breeding, and disease.

## 1. Introduction

Bactrian camels are mainly distributed in desert and semi-desert areas of western and northern Asia, and are strongly adapted to their ecological environment. They have a strong ability to survive due to their high temperature tolerance, drought tolerance, and salt tolerance, and can feed on prickly and alkaline plants [1,2]. Old World camels are typically multipurpose animals. In addition to their utilization for production as livestock (milk, wool, and manure) or slaughtered (meat, skin, and fat) animals, camels are valued for their power in different leisure and work activities (such as riding, packing, and carting) [3]. Estimates of the heritability, repeatability, and breeding values for meat yield, milk yield, growth measures, reproductive traits, and adaptive traits are lacking for camels, unlike for other animals. Camel resources have also been influenced by the development of modern means of transportation. The market for the consumption of camel hair, camel meat, camel milk, and other products is not perfect, and little attention is paid to the camel industry, resulting in a rapid reduction in existing populations. This has made it difficult to promote development in the camel industry, ultimately resulting in a sharp decline in the number of camels, causing their wild ancestors to be designated as critically endangered by the IUCN, and accelerating the depletion of camel genetic resources. China, Mongolia, Russia, and Kazakhstan are home to the world’s largest populations of Bactrian camels. At present, there are seven main breeds of Bactrian camel, and four of which are found in China, namely the Xinjiang Bactrian camel, Sunite Bactrian camel, Qinghai Bactrian camel, and Alashan Bactrian camel. Xinjiang Bactrian camels are divided into Tarim Bactrian camels (TLM) and Junggar Bactrian camels (ZGE) [4]. In 2011, the Chinese Commission of Animal and Poultry Genetic Resources identified TLMl and ZGE as genetic resources based on data collected during investigations and the relevant academic literature. According to statistics, there are about 130,000 Bactrian camels in Xinjiang, including 110,000 in Tarim and 20,000 in Junggar [5]. However, a growing demand for sustainable milk and meat production has reignited interest in dromedaries and Bactrian camels, making them a focus and challenge for animal nutritionists and disease researchers.

With rapid development in sequencing and bioinformatic technologies, these have been widely used in the study of population genetics, genetic mapping, and whole-genome selection breeding. In the study of genetic differentiation in cattle, horses, and sheep, different methods are selected according to different research purposes. Rapid development in DNA-based approaches, specifically sequencing methods such as GBS, has provided important information. This information includes species’ taxonomy, distribution, evolutionary history, genetic diversity, population genetic structure, conservation genetics, molecular breeding, and germplasm conservation [6,7]. The Bactrian camel is one of the livestock animals for which population genome sequencing has most recently been performed and for which genetic diversity has most recently been explored. It was not until 2012 that the world’s first Bactrian camel genome sequence map was drawn and analyzed [8]. In 2013, Burger and Palmieri used shotgun sequencing to describe SNPs and determine population parameters for heterozygosity in Bactrian camels [9]. In 2014, Wu et al. [10] revealed the mechanisms of evolution and adaptation in Bactrian camels, dromedaries, and alpacas using whole-genome sequencing and transcriptome sequencing. Chinese scholars have studied individuals, population structure, and the origin and evolution of camels using microsatellite markers, blood protein markers, mitochondrial DNA markers, Y chromosome genetic markers, and nuclear genome markers, among other methods. Unfortunately, these DNA markers can only cover a small amount of the camel genome, and only a small number of genetic variation data are available regarding the camel genome. Genome sequencing for camelids (Bactrian camels, dromedary camels, and alpacas) has now been completed, including for wild Bactrian camels. This work will provide rich resources and data for in-depth camel genome research [11]. In addition, some unique biological characteristics of camels may have a significant impact, with progress in biotechnology and in-depth research in the fields of livestock breeding, bionics, medical research, etc. [12,13]. Bactrian camels contain rich resources and a huge gene pool, which are expected to be further studied, along with their ability to adapt to extreme temperatures and their strong dehydration resistance. Therefore, it is necessary to conduct a comprehensive study on the genetic diversity, population differentiation, and degree of inbreeding of Bactrian camels in different regions at the genomic level.

However, economic factors (low economic benefit from raising camels), environmental factors (droughts), and the camels’ own biological factors (a slow breeding speed and long breeding cycle) have led to a serious decline in the quantity and quality of Bactrian camels [14]. This is the first problem to be solved at present. Another concern is sustainable development in the camel industry and the protection and utilization of germplasm resources. We therefore studied the population quality and genetic diversity of Tarim and Junggar Bactrian camels, and searched for candidate genes related to their biological characteristics to evaluate their molecular characteristics. The genetic diversity, population structure, and genetic relationships were analyzed using GBS sequencing technology. On this basis, the effects of resource conservation were evaluated according to differences between individuals or populations. The genetic variation and candidate genes for biological characteristics were revealed at the genome level. This also provides an important reference for determining valuable genetic resources and improving camel breeding. Furthermore, it will be helpful for research into economically important characteristics and the mechanisms underlying biological characteristics, as well as for carrying out molecular breeding and disease research on Bactrian camels.

## 2. Materials and Methods

### 2.1. Sample Collection

The experimental study was conducted in 2022. A total of 118 Bactrian camels were randomly selected from two conserved populations of Tarim Bactrian camels (TLM) and Junggar Bactrian camels (ZGE). Samples of TLM were collected from Keping County, Aksu, southern Xinjiang, and samples of ZGE were collected from Fuhai County, Altay, northern Xinjiang. (Detailed information is listed in Table 1.) The genetic relationship between individuals was unknown, and blood samples were collected. For sampling, 5 mL of blood was taken from the jugular vein of each individual Bactrian camel, transferred into an ethylene diamine tetra-acetic acid (EDTA) anticoagulant tube, and stored in a −80 °C freezer.

DNA was extracted from the blood samples using a blood genomic DNA extraction kit (Omega Bio-Tek # D3392-01, Shenggong Biotechnology Engineering (Shanghai) Co., Ltd., Shanghai, China), following the manufacturer’s protocol. The genome of Bactrian camels (*Camelus bactrianus*) (Biological Project Database: BCGSAC_Cfer_1.0) was used as the reference genome, and the BWA (version 0.7.15) software (parameters: mem-t 4-k 32-M) was used to align the valid paired-end data to the reference gene set and calculate the average alignment rate, average sequencing depth, and average coverage. The GATK standard process was used to detect the variation sites and obtain the site variation file.

### 2.2. Population Genetic Structure Analysis

The P-distances model in TreeBest software was used to calculate genetic distances and construct phylogenetic trees, with bootstrap values set to 1000 to test the reliability. Based on these results, a phylogenetic tree was constructed using the neighbor-joining method. A genome relationship G-matrix was created based on Van Raden’s method using genome-wide markers [15]. The genetic relationship of the populations was analyzed based on the G-matrix. Principal component analysis (PCA) was performed based on the SNPs using rMVP (version 1.0.4) software [16].

### 2.3. Population Genetic Diversity Analysis

The observed heterozygosity (Ho) and expected heterozygosity (He) were calculated using PlinkV1.90. The expected heterozygosity (HE), observed heterozygosity (HO), polymorphism information content (PIC), effective number of alleles, and minimum allele frequency (MAF) were used to analyze the genetic diversity.

The fixed coefficient F of the population reflects the level of allele heterozygosity in the population. The genetic differentiation index (Fst) of each SNP locus in all of the individuals was analyzed. Based on the high-quality SNP information obtained after screening, the PLINK software was used to detect the selection signal. The sliding nucleotide diversity (π) ratio and Fst were calculated using a 500 Kb window and 20 Kb step size. The top 5% of the π ratio and Fst values were used as thresholds to analyze the population selection signal, and the intersection of the top 1% was used as the candidate site. GO and KEGG enrichment analysis were carried out for the genes where the selected sites were located.

Genome-wide sequential homozygous fragment (ROH) analysis was conducted. The ROH-based inbreeding coefficient was calculated by calculating the total length of the ROH segments in an individual as a proportion of the total length of the autosomal genome. The ROH length was calculated for each sample using Plink (V1.90). The inbreeding coefficient of each individual based on the long homozygosity (ROH) was obtained by counting the ROHs of each individual; the inbreeding situation of the sample population was analyzed, and the genetic relationship was inferred.

## 3. Results and Analysis

### 3.1. Sequencing Data Output and Quality Control

GBS sequencing was performed on 118 Bactrian camel blood genomes whose parameters all met the sequencing criteria. In total, 327.52 Gb of raw data were generated, with an average of 806.69 Mb per genome. After filtering, a total of 327.49 Gb of valid data remained, i.e., 99.99% of the original reads, averaging 805.88 Mb per genome. The proportions of bases with Q20 quality ranged from 90.25 to 97.58%, with the average being 95.65%. The GC base contents ranged from 37.1 to 38.93%, with an average of 38.09%. The effective read lengths were 1.94~17.08 Mb, with 5.60 Mb being the average. The ratios of the effective reading lengths to the length of the reference gene were 89.02~95.71%, and the average ratio was 94.75%. The ratio of the total sequenced bases to the genome size, that is, the minimum sequencing depth, was 5.65×; the maximum sequencing depth was 31.64×, the average sequencing depth was 14.33×, and the average coverage was 3.57%. The GC distribution was normal, the samples were free of contamination, the sequencing quality was high, and the data were good.

### 3.2. SNP Detection and Quality Control

SNP site quality control was carried out to retain the best-quality typing sites for subsequent analysis. The specific quality control conditions and results are shown in Table 2.

### 3.3. Analysis of Population Genetic Structure and Genetic Relationship

#### 3.3.1. Population Phylogenetic Tree Analysis

**Figure 1:**
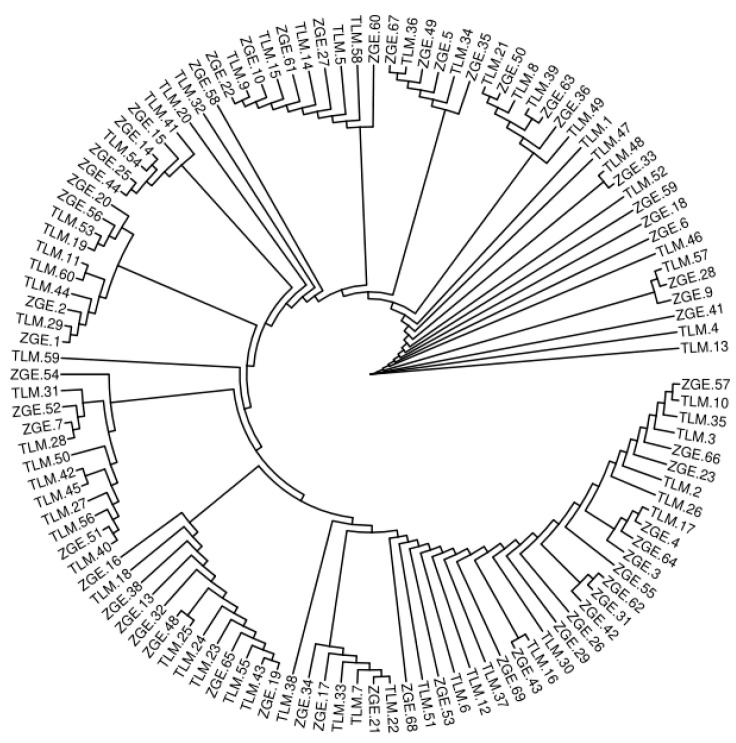
Phylogenetic tree of Tarim (TLM) and Junggar Bactrian camels (ZGE).

The phylogenetic tree provides insight into the genetic relatedness and ancestral relationships among individuals in the Bactrian camel population. The neighbor-joining method was employed to construct the phylogenetic tree. The results indicate that TLM and ZGE share a recent common ancestor, suggesting a close genetic relationship with a short evolutionary divergence time. The results of the phylogenetic tree analysis are shown in Figure 1 below.

#### 3.3.2. Principal Component Analysis

**Figure 2:**
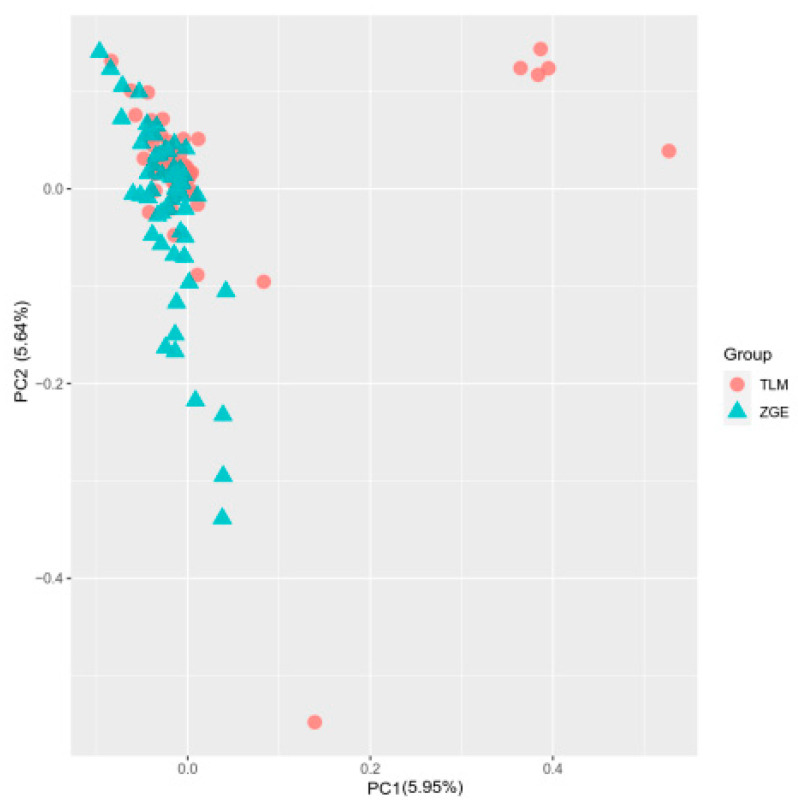
TLM and ZGE principal component analysis results. Note: The horizontal and vertical axes in the figure represent principal component 1 and principal component 2, respectively. Different colors indicate different groups: Tarim Bactrian camels (TLM) and Junggar Bactrian camels (ZGE).

The results of the principal component analysis indicate that the populations of TLM and ZGE can be divided into four clusters. The results of the first principal component analysis indicate that individuals from TLM and ZGE populations tend to cluster together, indicating high similarity between the majority of individuals from both populations. However, the results of the second principal component analysis indicate that a small number of individuals from TLM are more scattered; this suggests a lower similarity between these individuals and other individuals. Overall, the differentiation between the two populations is not pronounced, with only a few TLM individuals showing higher distinctiveness from all of the other individuals. The PCA results are shown in Figure 2.

#### 3.3.3. Analysis of Genetic Distance between Individuals

**Figure 3:**
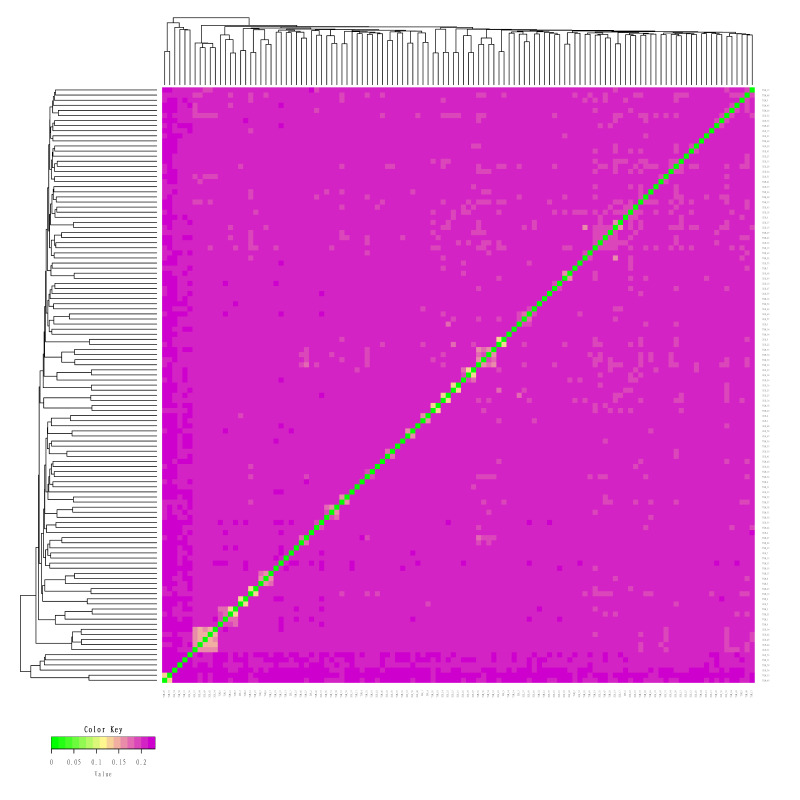
Visualization results for genetic distance analysis. Note: The graph shows the genetic distance between two individuals. The closer the color is to green, the closer the genetic relationship is. The abscissa and ordinate indicate the individual ID.

The genetic distance between individuals in the Bactrian camel population was analyzed using genetic markers or the similarity of alleles. The results indicate that TLM and ZGE have a close genetic distance. The results of the genetic distance analysis are shown in Figure 3.

### 3.4. Analysis of Genetic Diversity

#### 3.4.1. Statistical Analysis of Heterozygosity in Genomic Loci

The statistical analysis of the heterozygosity in genomic loci included the analysis of the effective population content (Ne), polymorphic marker fraction (PN), expected heterozygosity (HE), observed heterozygosity (HO), polymorphism information content (PIC), effective number of alleles, and minimum allele frequency (MAF). Effective population content (Ne) analysis was performed using the computational method proposed by Herrero-Medrano et al. (2013) and Sved [17]. The minimum allele frequency (MAF) for each locus was calculated using Plink software (version 1.9) [18]. The results of the genetic diversity analysis are shown in Table 3.

#### 3.4.2. ROH Statistics and Analysis

**Figure 4:**
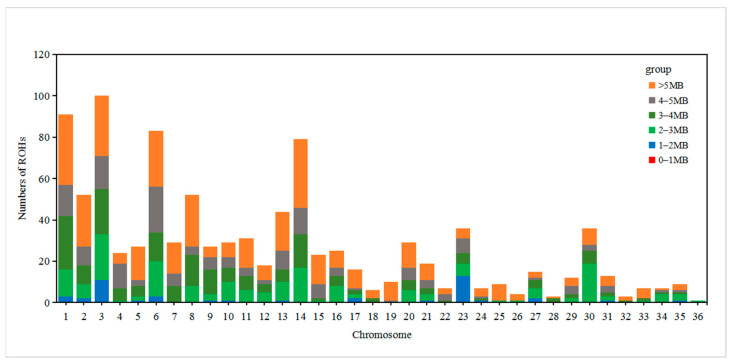
Distribution of ROH quantity on each chromosome. Note: the abscissa is the chromosome number, the ordinate is the number of ROHs, and the different colors represent the fragment sizes of the ROHs.

**Figure 5:**
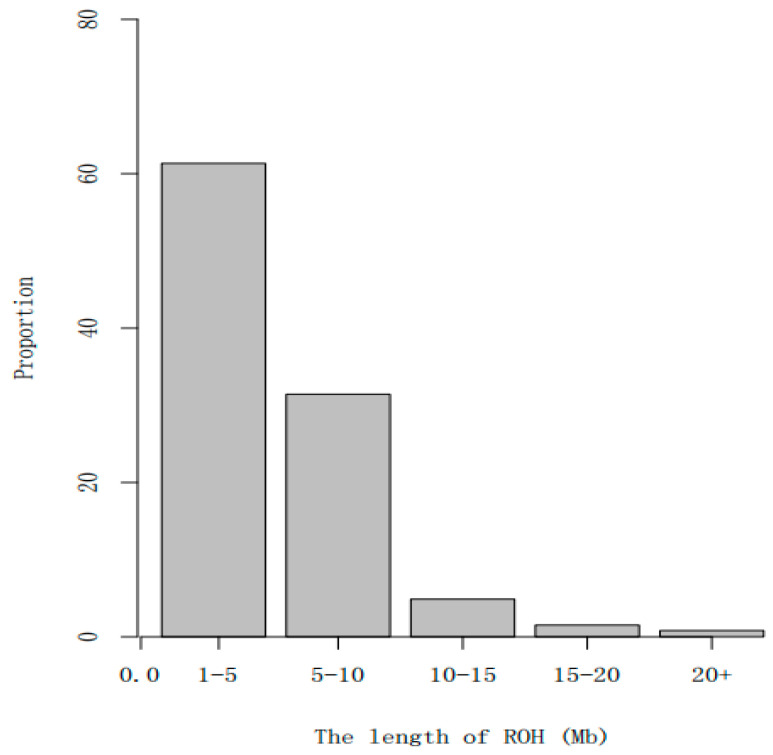
ROH length distribution of sample population. Note: the abscissa represents the length interval of the ROH, and the ordinate represents the population proportion.

**Figure 6:**
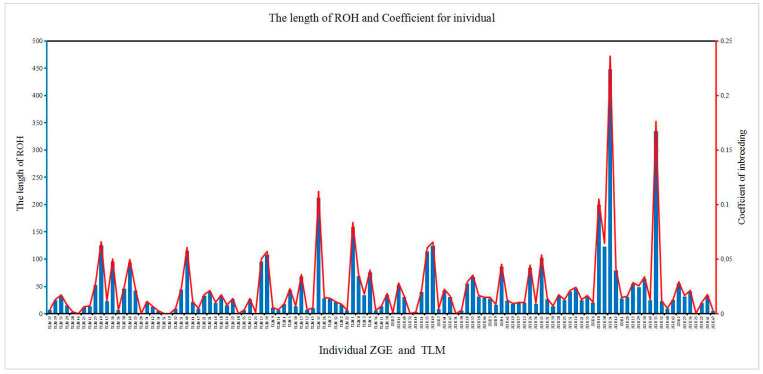
Distribution of sample individual ROH lengths. Note: the abscissa represents the individual Bactrian camels. The ordinate represents the length and individual coefficients of the ROH.

A long ROH may indicate a more recent relationship, and the greater the number of such segments, the higher the likelihood of inbreeding within the family. A short ROH indicates a distant relationship, and the more such segments there are, the less likely inbreeding is to occur within the family. The ROH length and number of ROHs were calculated for each sample using Plink (V1.90). In terms of ROH distribution, there were more ROHs on each of the chromosomes 1, 3, 6, and 14. The distribution results are shown in Figure 4. Most of the population had ROHs between 1 and 5 Mb. The distribution results are shown in Figure 5. The samples with ROHs of length between 0 and 50 Mb had the greatest number of individuals. The distribution results are shown in Figure 6.

#### 3.4.3. Analysis of Inbreeding Coefficient Based on ROH

**Figure 7:**
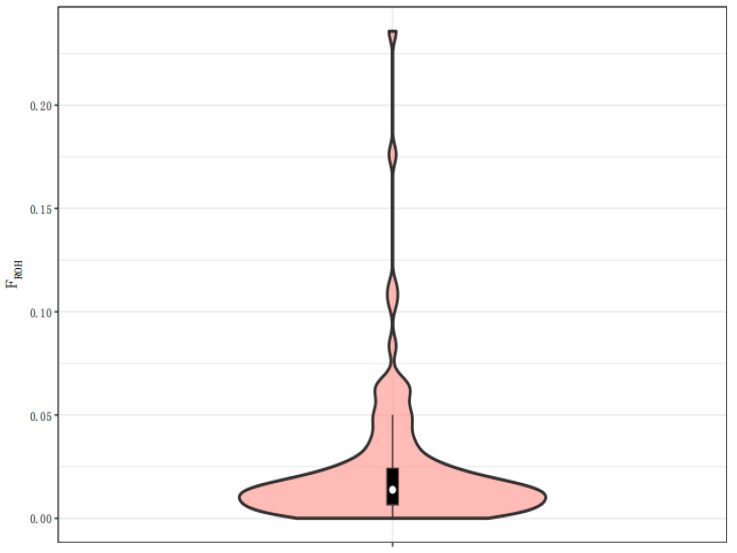
Distribution map of the inbreeding number FROH based on ROHs. Note: The violin plot is mainly used to display the distribution of the data. The white dot in the center represents the median of the population FROH, and the upper and lower edges of the middle black box are the upper and lower quartiles of the population FROH, respectively. The width of the violin graph represents the probability density distribution of the population FROH, and the wider the part of the violin graph, the greater the number of samples at that level, and vice versa.

The longer the total length or number of ROHs in an individual, the higher the inbreeding coefficient of the individual. The inbreeding coefficient value of each individual was obtained by counting the ROHs of each individual in the population. The proportions of samples corresponding to different inbreeding coefficients can be visualized, and are shown in Figure 7. The figure shows the distribution of the inbreeding coefficients for all of the samples. The average ROH inbreeding coefficient of this population was 0.023, indicating a low inbreeding level. The conservation effect of the two populations was good.

### 3.5. Population Selection Signal Analysis

#### 3.5.1. Nucleotide Polymorphism (π) Analysis

**Figure 8:**
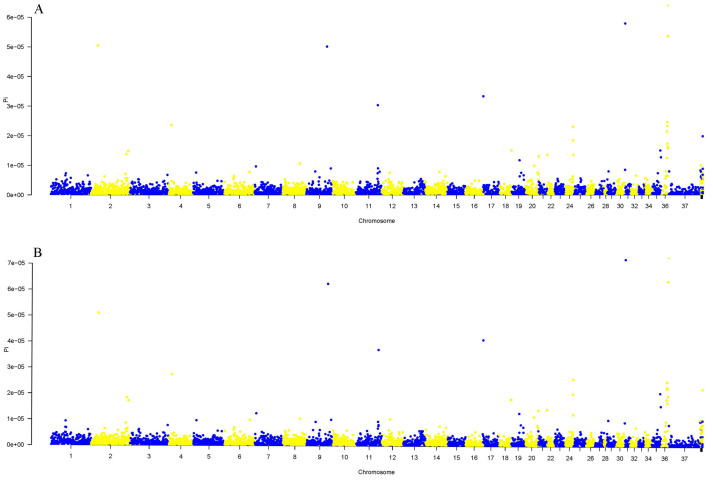
Results of the nucleotide polymorphism analysis of TLM and ZGE. Note: (**A**) is the Tarim Bactrian camels (TLM); (**B**) is the Junggar Bactrian camels (ZGE). The horizontal axis represents the position on the genome, and the ordinate represents the π value.

By sliding a 500 kb window across the genome, the differences in population genetic information (SNPS) in the sliding window were analyzed. The population π values reflect the genomic base diversity of the Tarim and Junggar Bactrian camel populations. The π analysis results are shown in Figure 8.

#### 3.5.2. Analysis of Population Differentiation Index (FST)

**Figure 9:**
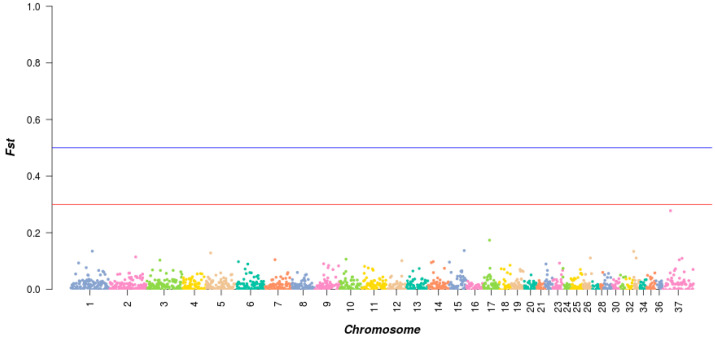
FST plot results for TLM and ZGE populations.

The genetic differentiation index is suitable for a comparison of diversity among subpopulations and is used to measure the degree of population differentiation. The red and blue lines represent the FST threshold values of 0.25 and 0.5, respectively. FST values range from 0 to 1, where a higher FST value indicates a greater genetic differentiation among populations. The results of the FST analysis of the Tarim (TLM) and Junggar Bactrian camels (ZGE) are shown in Figure 9.

#### 3.5.3. Selection of Signal Gene Enrichment and Annotation

**Figure 10:**
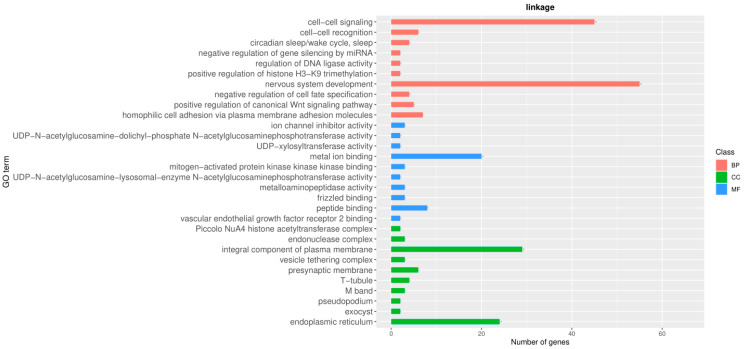
GO enrichment results for selected genes.

**Figure 11:**
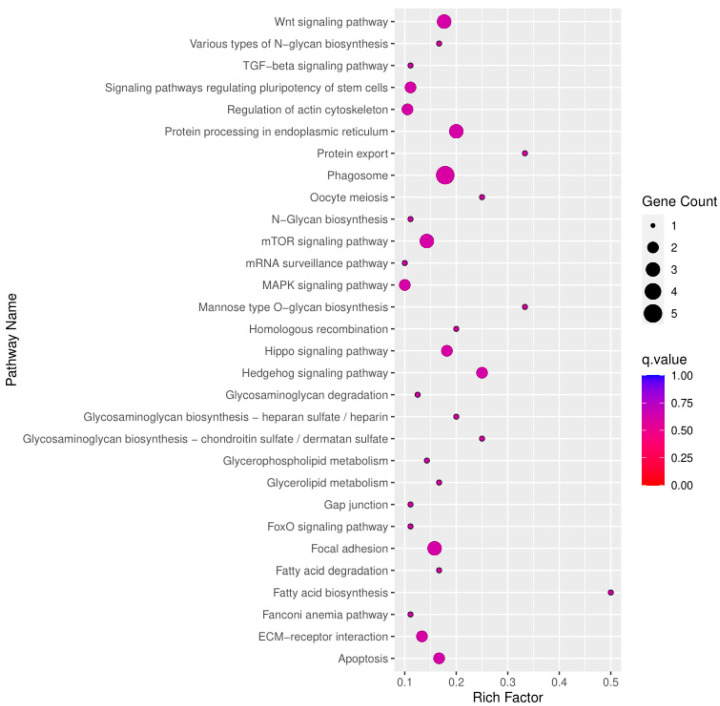
KEGG pathway enrichment results for selected genes. Note: Q value is the *p* value corrected for multiple hypothesis testing. The range of the q value is [0, 1]. The closer the value to zero, the more significant the enrichment.

Selection signal analysis was carried out according to the Fst and π between the TLM and ZGE populations, and the top 5% region with the largest Fst value was selected to be screened. The smallest 5% region in the nucleotide polymorphism analysis was also selected to be screened. Functional enrichment analysis was performed on the selected regional annotated genes from the TLM and ZGE populations. We took the intersection of the two parts of the selected region as the final selected region. A total of 1564 SNPs were screened from the selected regions, and 108 genes were selected for GO functional annotation analysis. These selected genes were mainly enriched in cell–cell signaling, cell–cell recognition, nervous system development, the negative regulation of cell fate specification, the positive regulation of the canonical Wnt signaling pathway, homophilic cell adhesion via plasma membrane adhesion molecules, and other biological processes. The main cell components involved were as follows: integral components of plasma membrane, presynaptic membrane, and endoplasmic reticulum. The main molecular functions involved included metal binding and peptide binding. The GO enrichment results are shown in Figure 10. These selected genes’ KEGG results, enriched for the top 30 pathways, were mainly concentrated in the Wnt signaling pathway, signaling pathways regulating the pluripotency of stem cells, the regulation of actin cytoskeleton, protein processing in the endoplasmic reticulum, phagosomes, the MAPK signaling pathway, apoptosis, and other regulatory pathways. The 30 most significant pathway results were enriched, as shown in Figure 11.

### 3.6. Cluster Analysis and Family Construction

**Figure 12:**
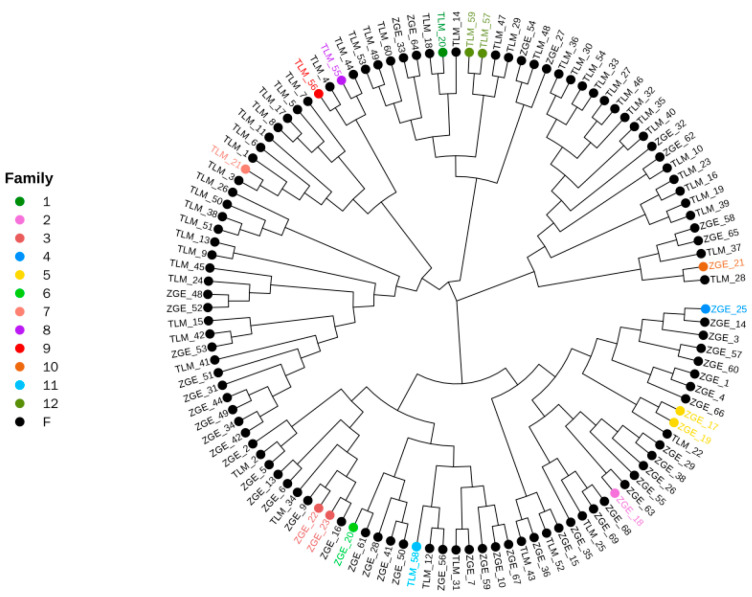
Cluster analysis results for the TLM and ZGE samples. Note: For the male camel samples marked with colors, one color represents one family. Tarim Bactrian camels (TLM) and Junggar Bactrian camels (ZGE).

This was performed due to the need for species conservation. Combined with the results of the genomic genetic relationship analysis and cluster analysis, the existing genetic relationships between female camels and male camels were used to divide them into different families. In total, 93 female camels were found to be far from the detected male camels, and so they were classified as “other”. The cluster analysis results of all of the Bactrian camel samples are shown in Figure 12.

## 4. Discussion

Bactrian camels are a rare and valuable genetic resource in northwest China, playing an important role in development in animal husbandry and ecological balance. This is the main motivation for the protection of this genetic resource, but there is also the promise of the implementation of scientific and effective measures for breed protection, as well as research into Bactrian camels’ genetic diversity and genetic structure. Genetic diversity is one of the key issues in the protection of Bactrian camel resources in this region. With rapid development in molecular biotechnology, sequencing-based technology has become mainstream, which makes it possible to study the genetic structure and genetic diversity of Bactrian camels at the genomic level. At the same time, the effects of efforts to protect Bactrian camels can be monitored more comprehensively and accurately. Therefore, in this study, the genetic diversity, genetic structure, genetic relationships, and degrees of inbreeding of 60 Bactrian camels from Tarim and 58 from Junggar were investigated using GBS. The sequencing results included 327.49 Gb of valid data, and 21,971 high-quality SNPs were obtained. The detected loci were distributed on each autosome, and the number of loci basically corresponded to the length of the chromosome, which indicated that the obtained variation information was representative. These data will provide a basis for the preparation of a breeding SNP microarray for Bactrian camels. The genetic relationships and inbreeding coefficients obtained from the study were based on analysis at the genome level, truly reflecting the actual conservation situation of the populations of TLM and ZGE, and providing a powerful technical means for the protection of germplasm resources and optimization of the selection and matching scheme.

### 4.1. Population Genetic Structure Analysis

Population structure is an important method for studying the evolutionary processes and relationships within a species. In order to evaluate, protect, and utilize the genetic resources of Bactrian camels scientifically, Han et al. [19] used restriction enzymes to analyze the restriction fragment length polymorphisms (RFLPs) of the mitochondrial genomes of domestic and wild camels in Gansu Province. Three different restriction bands of endonuclease were found between domestic and wild camels. This proved that the mitochondrial genome of Bactrian camels is inherited from the mother. The same is the case for other mammals. Research by other scholars has shown that the split between the ancestors of wild and domestic Bactrian camels was more recent, and it has been estimated to be at 1.1 (0.6–1.8) Mya [20,21]. Cheng Jia et al. [22] studied the partial sequences of the mitochondrial Cytb gene in domestic Bactrian camels and wild Bactrian camels. The results showed that the selected Bactrian camels from Alashan, Gansu, and Xinjiang were grouped into the same group. Therefore, it is speculated that domestic Bactrian camels are different from wild Bactrian camels, and that domestic Bactrian camels originated from the same maternal line. Therefore, we believe that the ancestors of Bactrian camels in China and the existing wild Bactrian camels are not of the same subspecies. China’s domesticated Bactrian camels are of a single maternal origin, and wild Bactrian camels are not the direct ancestors of domestic Bactrian camels. Jirimutu et al. [23] also used the mitochondrial Cytb gene sequence to confirm the existing idea that wild and domestic Bactrian camels have their own independent maternal origins.

In a study involving the full genome sequence data of six camels from the Arabian Peninsula and the genotyping-by-sequencing data of forty-four camels from Sudan, genome admixture and principal component analyses indicated distinct geographic separation between the camels from Sudan and those from the Arabian Peninsula [24]. However, there was no specific within-population genetic distinction. In a related study, microsatellites revealed a defined global structure, separating East African and South Arabian camel populations from those of North Africa, North Arabia, and South Asia [25]. In this study, the TLM and ZGE are domesticated Bactrian camels. The results of the genetic structure cluster analysis showed that the TLM and ZGE Bactrian camels were clustered into one group. Principal component analysis showed that most of the ZGE and TLM Bactrian camel populations were closely clustered together. Only a few of them were distant and scattered. The individuals of the ZGE Bactrian camels were relatively clustered. Some individuals of the TLM Bactrian camels deviated from the group. However, there was no obvious stratification between the two groups. With the contribution of the second principal component being relatively low, the separation of TLM and ZGE from other individuals is not evident when conducting analysis with all SNPs. However, both the phylogenetic tree and the analysis of genetic distances indicate that TLM and ZGE have a close genetic distance. This is similar to the results of Han et al., Cheng Jia et al., and Jirimutu et al. Population structure analysis, principal component analysis, and cluster analysis showed that TLM camels and ZGE camels may come from the same ancestor or maternal origin. On the other hand, Chen Huiling et al. [20] studied the origin and phylogenetic evolution of Bactrian camel sire lines in China. They studied male individuals of Hexi double peak, Qinghai double peak, Alashan double peak, Sun double peak, southern Xinjiang, and Xinjiang double-humped camels. The Y chromosome USP9Y, UTY, DBY, and SRY genes were partially sequenced to screen out Y-SNP polymorphic markers. With the help of Y chromosome molecular markers, it was found that the selected genes had the same haplotype and showed less differentiation among the groups. The results suggest that Bactrian camels in China have a single paternal origin.

The genetic differentiation, reproduction modes, and generation lengths among species are closely related. There are similarities among Bactrian camel populations in China, Mongolia, Iran, and Kazakhstan, which share several common haplotypes. This phenomenon does not seem to bear much relation to regional location. It is foreseeable that domestic camels may often travel between different geographical areas. TLM and ZGE Bactrian camels are selected via open sub-breeding. The use of superior non-closely related male camels in the generation breeding process will result in a high degree of genetic variation among individuals in the population. The results of the phylogenetic tree analysis and genetic relationship matrix of the TLM and ZGE Bactrian camels show that the genetic relationships and genetic distances between TLM and ZGE Bactrian camels are very different. The differentiation between TLM and ZGE Bactrian camels showed an unstable and chaotic state, with some crossbreeding and individual outliers, indicating that the genetic structure of TLM and ZGE Bactrian camels has changed greatly over time. It is speculated that this may have been caused by the small size of the conservation population.

### 4.2. Population Genetic Diversity Analysis

Abundant genetic diversity is the basis through which species cope with environmental changes. There are many genetic variations in camels that can be used to improve economically important traits. Bactrian camel breeds distributed in different ecological regions have formed different characteristics and potential development value. The evolutionary relationships and genetic diversity of Bactrian camels have been studied. Ishag et al. (2010) reported the SNP 419 C>T in the non-coding region (intron 1) of the GH gene of Kenani, Lahwee, Rashaidi, Anafi, Bishari, and Kabbashi camel breeds of Sudan. This could aid in the determination of genetic relationships among the different camel breeds [26]. Zhang Dongcheng et al. [27] found that the genetic diversity of the Cytb gene in Chinese Bactrian camels was rich, and the evolution of the D-loop sequence was in line with neutral selection according to sequencing and analysis. The study included southern Xinjiang, northern Xinjiang, eastern Xinjiang, Qinghai Bactrian, Hexi Bactrian, Alxa Bactrian, and Sunit Bactrian camels in China. The domestic Bactrian camels clustered into a clade, and wild Bactrian camels, dromedary camels, and alpacas formed different, independent clades. Wang Le et al. [28], through a UPGMA phylogenetic tree based on Da genetic distance, showed that the domestic Bactrian camels in the five regions could be divided into two branches. Alashan Bactrian, Hexi Bactrian, and Xinjiang eastern Bactrian camels were clustered into one branch, and only ZGE and TLM Bactrian camels were clustered together. It is speculated that this result may be due to the mountains in the north of Xinjiang and the desert in the middle of the two groups of ZGE and TLM Bactrian camels isolated from the other three groups; so, Xinjiang Bactrian camels form an independent branch with rich diversity.

According to seven microsatellite markers, the genetic diversity among the Ourdhaoui Médenine, Ourdhaoui Tataouine, and Merzougui camel subpopulations in Tunisia revealed a mean number of alleles (MNA) of 6.5. The expected heterozygosity (He) ranged from 0.76 to 0.84, while the observed heterozygosity (Ho) was 1.0. The subpopulations showed a moderate genetic structure based on the fixation index (FST = 0.052) [29]. Four dromedary populations—Guerzni, Harcha, Khouari, and Marmouri (Morocco)—were studied using 16 microsatellite markers; all of the loci were polymorphic, while the MNA was 13.4. The He ranged from 0.702 to 0.748, while the highest value of Ho was 0.699. The lowest genetic distance showed that the camels were well diversified [30]. The mean observed heterozygosity (Ho), mean expected heterozygosity (He), and polymorphism information content (PIC) of the TLM and ZGE Bactrian camels were 0.235, 0.245, and 0.202, respectively. The two populations belonged to a moderately heterozygous population. The average observed heterozygosity was close to the average expected heterozygosity, which indicated that both Tarim and Junggar camels had high purity and abundant genetic diversity. Most of the individuals of the TLM and ZGE Bactrian camels were clustered, and a small number of individuals were scattered, which indicated that the conservation effect of TLM and ZGE was almost perfect, and some individuals with pure blood appeared in the population. Given their typically multipurpose use and the weak anthropogenic selection pressure, their phenotypic diversity means that they are mostly distributed into different eco-types, rather than breeds, with classification mainly being based on the geographical distributions of ethnic groups and pastoral communities [31]. It is quite possible that some biological and economic characteristics are already fixed and can be transmitted steadily. However, it should be noted that the data for TLM and ZGE Bactrian camels seem to indicate that there is a crossover phenomenon between the two populations in different geographical locations, and the differences between the populations are not related to geographical locations. The average inbreeding coefficient (FROH) of TLM and ZGE Bactrian camels was 0.023. The low inbreeding level indicated that TLM and ZGE Bactrian camels were effectively protected in the relatively isolated geographical environment, and the two populations were not affected by exotic species or inbreeding. However, the TLM and ZGE populations were clustered into the same clade. Consider that, for thousands of years, Bactrian camels have been an important means of transport in desert areas. It is possible that, for a long time in the past, frequent introduction events or a large number of trading activities between different markets occurred in different regions, resulting in gene exchange between Bactrian camels in different geographical locations. This may have resulted in TLM and ZGE Bactrian camels gradually coming to belong to the same branch.

### 4.3. Selective Signal Analysis

Recently, there has been increased interest in the field of camel immunology. Bactrian camels are also a valuable genetic resource, with a rich gene pool that may be the perfect answer to adapting to harsh climatic conditions and efficiently dealing with the challenges that they present. Bactrian camels are highly adapted to the extreme desert ecosystem, with higher resistance to a wide range of pathogens compared to many other species from the same geographical region [32,33]. Research shows that higher mean heterozygosity (0.560) was found for Arabian Peninsula camels compared to the value of 0.347 for the Sudanese. Pooled heterozygosity analysis revealed 176, 189, and 308 candidate regions under positive selection in Sudanese camel populations [24]. Xinjiang camels have the highest nucleotide polymorphism, the fastest decay rate of the LD coefficient, and the highest genetic diversity [34]. TLM and ZGE Bactrian camels have been formed due to long-term natural and artificial selection in special geographical areas and ecological environments. Therefore, it is necessary to study the genetic diversity, genetic structure, and genes that play important roles in the evolution process. GO and KEGG analyses were performed on 108 strongly selected genes in TLM and ZGE Bactrian camels. GO enrichment showed that these genes are widely involved in biologically related processes, such as energy regulation and metabolism, growth and development regulation, and stimulus response. The KEGG was mainly concentrated in the Hedgehog signaling pathway, Wnt signaling pathway, the regulation of actin cytoskeleton, apoptosis, glycerolipid metabolism, the cell cycle, and other pathways. The salt tolerance and drought tolerance of Bactrian camels may be related to these pathways. Studies have shown that fat metabolism is closely related to the Hedgehog (Hh) signaling pathway, and Hh signaling is important for lipid metabolism. Conversely, lipid metabolism is also critical for Hh signaling [35]. The Hh signaling pathway is critical in controlling many processes involved in vertebrate embryonic development and adult homeostasis, including tissue/organ patterning, cell proliferation and differentiation, stem cell maintenance, and tissue repair after injury [36]. Wnt signaling is an evolved intercellular coordination mechanism that is central to many developmental and disease-related processes. It is remarkably conserved throughout the metazoan lineage and is involved in a variety of physiological processes, such as stem cell regeneration, proliferation, division, migration, cell polarity, and specification, which determines cell fate, neural crests, neural symmetry, and morphogenesis [37]. The dysregulation of Wnt signaling has been linked to developmental defects, cancer, and degenerative diseases [38]. Studies have shown that Wnt/β-catenin signaling not only coordinates developmental processes and tissue homeostasis, but is also a key negative regulator of preadipocytes’ differentiation into adipocytes. Wnt1 and Wnt10b are involved in mediating adipogenesis as components of Wnt signaling transduction [39]. The cell surface antigen CD14 acts as a co-receptor for Toll-like receptors (TLRs), activating and enhancing innate immune responses to pathogens and tissue damage in macrophages and monocytes [40]. The forkhead box 0 (FoxO) family of proteins plays a critical role in mediating the insulin and insulin-like growth factor pathways involved in cell differentiation, apoptosis, and energy metabolism. The FoxO protein not only plays a role in glucose metabolism, but also participates in the regulation of lipid metabolism. It participates in mediated proteolysis, degrading and recycling damaged organs and proteins in cells to resist external stress [41]. The FoxO protein, as a downstream protein of the insulin pathway, may be the key molecule linking insulin resistance and lipid metabolism disorders, and may regulate the balance of blood glucose in the body [42]. The FoxO protein may become a new target for the clinical prevention and treatment of lipid metabolism disorders.

In addition, there is another important consideration. Camel milk (CM) is known for its benefits in the human diet and for health. Milk traits are very important in camel breeding, considering that, on average, the amount of milk produced by camels is six times greater than that produced by the indigenous cattle in drylands. Camel milk has some health benefits attributable to the camels’ consumption of high-quality desert plants. Camel meat is also highly valued for its nutritive and healthy contents [27,43]. Related research on camel milk has shown that it is low in sugar, low in cholesterol, high in minerals, high in vitamins, and high in insulin [33]. Camel milk (CM) has been found to have several health benefits, including antiviral, antibacterial, antitumor, antifungal, antioxidant, hypoglycemic, and anticancer effects. In addition, CM can counter signs of aging and may be a useful naturopathic treatment for autoimmune diseases. The composition of CM varies with geographic origin, feeding conditions, seasonal and physiological changes, genetics, and camel health status [44]. Alpha-lactalbumin, beta-casein, and vitamin C may be involved in alleviating oxidative stress and play an important role in reducing or inhibiting the production of reactive oxygen species (ROS), hydroxyl radicals, nitric oxide (NO), superoxide anions, and peroxy radicals. Lactoferrin appears to be a common anti-inflammatory bioactive ingredient in camel milk (CM). CM has higher levels of protective proteins (e.g., lysozyme, IgG, and secretory IgA) compared to cow’s milk, and the insulin-like protein activity of beta cells appears to be responsible for the immunomodulatory properties of CM [45]. Physical interactions between the human insulin receptor (hIR), insulin receptor signaling protein (IRS1), and growth factor receptor binding protein 2 (Grb2) are associated with hypoglycemic activity [46]. The health benefits of camel milk have been described for a variety of diseases such as diabetes, kidney disease, and hepatitis. Camel milk has a therapeutic as well as a preventive role in the maintenance of and improvements in the metabolic regulation of the body [45]. In a study of the effects of active ingredients extracted from whey on human cancer cells, the active antitumor component TR35 isolated from camel milk significantly inhibited the proliferation of Eca109 cells and induced their apoptosis. TR35 can inhibit the growth of xenograft tumors in nude mice without weight loss [47]. Anaerobic eubacterium (*Oontomyces* sp. CR2) isolated from the rumen contents of Bactrian camels has the highest bioconversion potential for lignocellulose, thus indicating that the rumen of Bactrian camels may be a good source for isolating anaerobic fungi in industrial applications [48]. These results indicate that Bactrian camels have a rich gene pool, and these genes could be studied as candidate genes that affect camel milk quality and stress resistance. In addition, there are still many selected functional genes to be annotated. In this study, the germplasm resources of TLM and ZGE Bactrian camels were explored at the genomic level, the conservation effect of the conserved population was analyzed, and the selected functional genes were screened. It is very significant to fully understand the strong adaptability of Bactrian camels and harness their excellent economically useful traits.

## 5. Conclusions

In order to utilize the potential of camels, there is a need for genetic improvement using marker-assisted selection, while sustaining the camels’ genetic diversity and resilience. In this study, GBS was used for the analysis of population structure, genetic diversity, and gene selection signals. The results show that the genetic resource conservation effects of the TLM and ZGE Bactrian camel populations were clear, and they had high nucleotide diversity and rich genetic diversity. TLM and ZGE have a close genetic relationship, and there has been obvious genetic exchange between them in the past for a long time. After years of geographic isolation, the degree of genetic exchange has decreased. The genetic variation information for Bactrian camels and 108 candidate genes related to Bactrian camels’ biological characteristics were obtained at the genome level, such as WNT1, WNT10B, FOXO6, CD14, SEC61A2, DPAGT1, etc. These findings will provide a basis for determining the mechanisms underlying important economic traits and biological characteristics, and prove useful for molecular breeding and disease research in Bactrian camels, particularly TML Bactrian and ZGE Bactrian camels.

## Figures and Tables

**Table 1:** Statistics of collected samples.

Population	Age (Year)	Sample Number	Male	Female
Tarim Bactrian camel (TLM)	2~5	60	7	53
Junggar Bactrian camel (ZGE)	2~5	58	5	53
Total	-	118	12	106

**Table 2:** Statistical description of SNP quality control.

Quality Control Standard	Number of SNPs
Total number of SNPs	34,367
SNP with MAF < 0.01	1
SNP not in Hardy–Weinberg equilibrium (*p* < 10^−6^)	880
SNP with call rate < 0.90	10,120
SNPs on chromosome X	668
SNPs used after quality control	21,971

**Table 3:** Population genetic diversity analysis statistics.

Title	Total	ZGE	TLM
Effective Population Content (Ne)	46.0	45.7	46.3
Polymorphic Marker Fraction (PN)	0.732	0.714	0.750
Expected Heterozygosity (HE)	0.245	0.216	0.273
Observed Heterozygosity (HO)	0.235	0.231	0.237
Polymorphism Information Content (PIC)	0.202	0.197	0.208
Effective Allele Number (EN)	1.307	1.304	1.311
Minimum Allele Frequency (MAF)	0.173	0.168	0.175

## Data Availability

All of the data generated or analyzed during this study are included in this published article.
